# Contemporary Preoperative Detection of Extraprostatic Extension in Prostate Cancer

**DOI:** 10.3390/cancers18030456

**Published:** 2026-01-30

**Authors:** Jan Stępka, Tomasz Milecki, Jędrzej Ksepka, Anna Kujawska, Jaśmina Hendrysiak, Wojciech A. Cieślikowski

**Affiliations:** 1 Doctoral School, Poznań University of Medical Sciences, 60-812 Poznań, Poland; 2 Department of Urology, Ministry of Internal Affairs Hospital, 61-701 Poznań, Poland; 3 Department of Urology and Oncological Urology, Poznań University of Medical Sciences, 61-701 Poznań, Poland; 4 Students’ Society of Urology, Poznań University of Medical Sciences, 60-812 Poznań, Poland

**Keywords:** extraprostatic extension, prostate cancer, multiparametric MRI, radiomics, machine learning, deep learning, nerve sparing surgery, side specific prediction, explainable artificial intelligence, surgical planning

## Abstract

Extraprostatic extension (EPE) is an important prognostic factor in prostate cancer and influences nerve-sparing decisions during radical prostatectomy. Multiparametric MRI (mpMRI) is the standard for local staging, but its sensitivity for EPE remains limited, and its interpretation is subject to inter-reader variability. In this narrative review, we aim to create an overview of contemporary strategies for the preoperative detection of EPE. We searched PubMed, Embase, Web of Science, and Google Scholar, focusing on studies published between 2015 and 2025 including articles evaluating clinical parameters, mpMRI features, nomograms, radiomics, machine learning, and deep learning models for EPE prediction. The analyzed literature was compared with respect to diagnostic performance, validation strategy, and clinical applicability of individual methods. Clinical parameters and traditional nomograms provide moderate accuracy for EPE detection. mpMRI improves staging, with tumor–capsule contact length as the most important single imaging marker. Radiomics-based and machine-learning models matched and occasionally outperform conventional approaches, achieving AUC values ranging from 0.75 to 0.85. Deep-learning models demonstrated similar performance by directly analyzing imaging data, although most lacked external validation and were sensitive to dataset heterogeneity. Several radiomics and deep learning models demonstrated performance comparable to, and in selected studies exceeding, expert radiologist assessment. Binary EPE classification has limited clinical value, while side-specific and graded EPE assessment offers a more clinically relevant approach. Translation of these tools into routine practice will require multimodal, side-specific, and externally validated models supported by automated segmentation and explainable artificial intelligence frameworks.

## Simple Summary

Extraprostatic extension occurs when prostate cancer grows beyond the prostate capsule and is an important factor influencing surgical strategy and complication rates. Standard tools, such as clinical parameters, risk calculators, and multiparametric MRI, help estimate this risk; however, their accuracy is limited and varies between observers. New artificial intelligence techniques are increasingly being explored to improve preoperative detection. Radiomics and deep-learning models can analyze subtle imaging patterns that are often invisible to the human eye and may support more personalized clinical decisions. This review provides a contemporary overview of current and emerging methods for detecting extraprostatic extension and discusses future directions of prostate cancer management.

## 1. Introduction

Extraprostatic extension (EPE) of prostate cancer is a critical factor influencing various outcomes following radical prostatectomy. EPE has been associated with increased rates of positive surgical margins [1,2], biochemical recurrence [1,3], poorer progression-free survival [2,3], and a more aggressive form of cancer [1]. In addition, a wider extent of EPE is associated with a worse prognosis for the patient [3].

Given its strong prognostic implications, the accurate preoperative detection of EPE is essential not only for oncologic risk stratification but also for optimizing the balance between cancer control and functional preservation during surgery. In cases where EPE is confidently excluded, nerve-sparing prostatectomy may be safely performed, preserving erectile and urinary function [4,5,6]. Excluding EPE on one side of the prostate is already highly beneficial, as it enables nerve-sparing surgery on the unaffected side, which significantly increases the likelihood of postoperative erectile function recovery compared with non-nerve-sparing surgery [7]. Conversely, when EPE is suspected or confirmed bilaterally, wide excision or bilateral neurovascular bundle resection is required to achieve negative surgical margins [8], which reduces the chances of erectile function recovery to a minimum [7]. Hence, overtreatment due to false-positive findings of EPE can lead to unnecessary loss of function, while undertreatment increases the risk of recurrence. Therefore, the preoperative identification of EPE has direct and important implications for surgical strategy.

Traditionally, clinicians have relied on a combination of clinical factors like PSA, biopsy Gleason score, number of positive cores, and nomograms like MSKCC [9] and Partin tables [10] to estimate the risk of EPE. Then, multiparametric MRI (mpMRI) has become a cornerstone in the preoperative assessment of prostate cancer extension beyond the capsule, as mpMRI became a gold standard in predicting prostate cancer [11,12] with dedicated scoring systems and radiological features, also indicating possible EPE [13]. However, clinical tools have their limitations, like poor sensitivity for the detection of EPE on MRI [14]. The emergence of machine learning (ML) and deep learning (DL) approaches offers the potential to enhance predictive accuracy by learning complex patterns from large, multimodal datasets [15,16,17]. These tools may bridge the gap left by conventional methods and provide more individualized, reproducible, and scalable risk stratification for EPE.

This narrative review aims to provide an overview of the current methods used to detect EPE prior to radical prostatectomy. We explore traditional clinical predictors and biopsy-based nomograms, assess the diagnostic performance and limitations of mpMRI and MRI-based grading systems, and examine the contribution of quantitative imaging features and radiomics. Particular focus is placed on emerging machine learning and deep learning models, which hold promise for improved detection. Additionally, we discuss future research directions. Through this review, we aim to highlight both the current capabilities and unmet needs in the preoperative evaluation of capsular extension in prostate cancer.

## 2. Materials and Methods

Relevant literature for this narrative review was searched using major biomedical databases, including PubMed, Web of Science, Google Scholar, and Embase. Articles published between 2015 and 2025 were considered to reflect both foundational and state-of-the-art developments in EPE prediction. The search strategy involved combinations of the following keywords and MeSH terms: “*prostate cancer*”, “*extraprostatic extension*”, “*EPE*”, “*MRI*”, “*radiomics*”, “*machine learning*”, “*deep learning*”, “*black-box’*, “*explainable AI’*, “*CNN*”, “*Convolutional Neural Network*”, “*capsular invasion*”, “*preoperative staging*”, “*EPE*”, “*extracapsular extension*”, “*ECE*” “*capsular invasion*”, “*radical prostatectomy*”, “*prostate mpMRI*”, “*PIRADS*”, “*artificial intelligence*” *and* “*MRI prediction models*”, “*nomogram*”, “*side-specific*”, “*nomogram validation*”.

Only original studies, reviews, and technical validation papers written in English were included. The included studies spanned a range of methodologies, including clinical risk calculators, MRI-based grading systems, quantitative MRI features, radiomics, and deep learning models trained on multi-institutional or single-center datasets. Priority was given to papers addressing the diagnostic performance of mpMRI, radiomic signatures, and artificial intelligence (AI)-based models in detecting EPE. Additional articles were identified through manual reference tracking and expert recommendations.

Artificial intelligence approaches were categorized into two groups. The first group comprised radiomics-based machine learning models. These workflows were defined as pipelines in which handcrafted quantitative features such as shape descriptors, first-order intensity statistics, and texture features were extracted from segmented regions of interest including the tumor, the tumor–capsule interface, or the whole gland, and were subsequently used as inputs to classical machine learning classifiers such as support vector machines, random forests, and gradient-boosted decision trees including XGBoost. The second group comprised deep learning models. These were defined as end-to-end or semi end-to-end convolutional neural network approaches that learn imaging representations directly from magnetic resonance imaging volumes or image patches, optionally preceded by automated segmentation.

## 3. Review

### 3.1. Parameters Used for EPE Detection

#### 3.1.1. Clinical Parameters: PSA Level, PSA Density, ISUP Grade Group, Positive Biopsy Cores

Prostate-specific antigen (PSA) remains one of the most widely used serum biomarkers in the diagnostic and risk stratification pathway of prostate cancer. Elevated PSA levels, particularly when combined with PSA density (PSAD), may indicate a higher risk of adverse pathological features, including EPE [18,19]. Research shows that PSAD is an independent predictor of adverse pathologic stage and PSA-free survival [20].

Biopsy-derived factors, such as the Gleason grade group and the number or percentage of positive biopsy cores, are also strong predictors of cancer aggressiveness and extent. This was confirmed by a study in which patients with EPE, compared to those without EPE, had significantly higher PSA levels and higher International Society of Urological Pathology (ISUP) grade group in biopsy, but there were no differences in the percentage of positive biopsy cores [21]. However, other studies support that the percentage of positive biopsy cores is higher in patients with EPE, while PSA levels and ISUP grade group do not differ significantly between those with and without EPE [22]. Furthermore, the available literature confirms that including PSAD, tumor percentage, and tumor length in the Partin tables would improve EPE prediction [23].

Considering the TNM (Tumor, Node, Metastasis) classification in prostate cancer staging, the occurrence of EPE classified as at least T3a corresponds to the high-risk group, regardless of the PSA level or ISUP grading on the three-tier prognostic scale (low, intermediate, high) included in the European Association of Urology (EAU) guidelines [24]. Some authors recommend using the five-tier Cambridge prognostic scale to improve the prognosis of cancer-specific mortality in primary non-metastatic prostate cancer [25,26]. According to the above scale, EPE occurs in risk group 4 together with Grade Group 4 and PSA > 20 ng/mL [25,26].

Moreover, in patients with PSA < 10 ng/mL, in addition to PSAD and Gleason score, lymphovascular invasion is a statistically significant prognostic factor for EPE [27]. Another preoperative predictor of EPE may be perineural invasion at needle biopsy, as evidenced by available publications [28,29].

However, some authors suggest that the detection of EPE during prostate biopsy is rare and should not exclude radical local treatment [30,31,32]. Taken together, PSA-based markers, particularly PSAD, along with biopsy-derived features such as ISUP grade group, tumor burden, and lymphovascular or perineural invasion, provide important but sometimes inconsistent information on the risk of EPE. Although EPE automatically upstages disease to at least T3a and places patients in a high-risk category, the variability of individual predictors highlights the need for their combined use within refined prognostic systems rather than reliance on any single preoperative parameter. The summary of clinical parameters used in EPE prediction is depicted in Table 1.

#### 3.1.2. The Role of Magnetic Resonance Imaging

Before the era of multiparametric magnetic resonance imaging, men with elevated PSA levels were referred for transrectal ultrasound-guided prostate biopsy. The guidelines changed after the PROMIS study, in which clinically significant prostate cancer (csPC) detection increased by 18% as a result of the performance and interpretation of mpMRI before the first set of biopsies [11]. Therefore, since 2019, this has been included in the official EAU guidelines and has been regarded as a gold standard diagnostic tool. Using mpMRI, it is now possible to detect the presence of EPE on a large scale. The inevitable step was the creation of standardized scales to unify radiological descriptions. One such scale is the PIRADS v2.1 scale, which assesses the likelihood of csPC based on mpMRI. The features visible in mpMRI that indicate EPE with the highest sensitivity and specificity include, among others, breach of the capsule with direct tumor extension and a tumor–capsule interface greater than 10 mm [33]. Depending on the source, the sensitivity and specificity of EPE detection based on mpMRI are 40.4–47.4% and 85.7–96.6%, respectively [34,35]. In addition, the ESUR scoring system shows moderate diagnostic effectiveness in detecting EPE [36]. Schieda, N. et al. [37] reported that the PIRADS scale can reduce differences in EPE detection accuracy caused by the lesser experience of the describing radiologist, simultaneously increasing sensitivity without reducing specificity. However, there are reports of high specificity but poor and heterogeneous sensitivity for local PC staging in MRI [38]. Parameters improving sensitivity with a simultaneous decrease in specificity for the detection of EPE are: tumor size, capsular contact, and ADC entropy [39]. Another useful tool is the 5-point Likert scale, which effectively determines the grade of EPE and seminal vesicle invasion (SVI) in mpMRI [40,41]. There is evidence that the maximum tumor diameter of Likert 3–5 lesions on MRI is an independent prognostic factor for EPE [42]. Additionally, Mehralivand EPE grade has the same diagnostic performance as the Likert scale, predicting both EPE and biochemical recurrence-free survival with a comparable degree of observer dependence [21,43].

An important aspect in EPE detection is the phenomenon of intrareader variability—a situation in which two different radiologists evaluate the same MRI scan differently. The literature data indicate that the consistency in detecting index lesions based on the PI-RADS v2.1 scale by radiologists with different levels of experience is substantial, while the consistency in excluding index changes is excellent regardless of the level of experience—85.1% for dedicated radiologists and 82.0% for non-dedicated radiologists [44]. In addition, clinically significant parameters for radiologist consistency, while minimizing intrareader variability, are PSAD ≥ 0.15 ng/mL/cc, pre-MRI high risk for PC, positivity threshold of PI-RADS score 4 + 5, PZ lesions, and homogeneous signal intensity of the PZ [44]. Also, reevaluation of the same image by another specialist improves EPE detection [45]. Therefore, it is reasonable to use the EPE grading system and artificial intelligence algorithms that estimate EPE risk without intrareader variability [46]. Existing EPE markers are effective regardless of tumor location, even in anterior prostate cancers (APCs), and the arc-dimension ratio may be a new marker for APCs [47]. Overall, mpMRI has become a cornerstone of preoperative EPE assessment, providing high specificity through standardized scales, but its sensitivity remains heterogeneous and dependent on reader experience. These limitations underline the need to focus on concrete, reproducible morphological mpMRI parameters and systems immune to intrareader variability.

#### 3.1.3. mpMRI Parameters Used in EPE Prediction

Several morphological features on mpMRI have been identified as potential predictors of EPE and mEPE—Table 2. Among these, the length of tumor–capsule contact is one of the most widely studied and reproducible metrics. Thresholds ranging from >10 to >20 mm have been associated with increased risk of EPE, though exact cutoffs vary between studies [33,48,49]. Some authors claim that the ISUP grade group influences the relationship between tumor capsule length and EPE, and that 15–16 mm can be considered the threshold value [50]. Other predictive signs include capsular bulging, irregularity, focal capsular disruption (“breach”), and extraprostatic protrusion into periprostatic fat. Some studies also consider asymmetry or obliteration of the neurovascular bundle (NVB) and the rectoprostatic angle [51]. These features, while helpful in identifying gross capsular extension, often fail to capture subtle or microscopic invasion, which does not result in observable changes in capsular contour. Bulging is a highly sensitive feature—81%, while macroscopic extension has a specificity of 100% [52]. In addition, the prediction of EPE can be improved by the capsular enhancement sign. When comparing MRI sequences, the literature refers to DCE-MRI as having superior performance in predicting EPE compared to other sequences [53]. Another useful parameter for predicting EPE is tumor contact area 1 (TCA1), which describes tumor dimensions across two planes, and tumor contact area 2 (TCA2), which describes the tumor’s contact area within the MRI volume. Although TCA1 and TCA2 do not show any advantage over tumor contact length (TCL), they are still useful parameters, especially in cT2N0M0 PC [54]. The EPE number in combination with radial distance is also an effective measurement in predicting biochemical recurrence and substaging of pT3a prostate cancer [55]. The above evidence indicates that there is no ideal parameter for the definitive detection of EPE. The solution seems to be the use of various parameters together with clinical assessment by specialist radiologists in order to maximize the chances of a correct diagnosis.

#### 3.1.4. Normograms

Normograms are tools used to assess the risk of EPE based on individual clinical parameters of the patient, mainly before planned radical prostatectomy. Traditional nomograms such as the Partin tables, the Memorial Sloan Kettering Cancer Center (MSKCC) nomogram, and the Cancer of the Prostate Risk Assessment (CAPRA) score are based on clinical parameters: PSA value, biopsy Gleason score, cT stage, and do not take into account mpMRI image, globally predicting EPE risk [9,56,57]. The tools that use MRI images are the Martini, Nyarangi–Dix, Soeterik, and Wibmer nomograms, which grant them the advantage of being side-specific—Table 2 [58,59,60,61]. They are especially important when deciding whether to perform a nerve-sparing radical prostatectomy on a certain side. Both traditional and MRI-inclusive nomograms have a moderate (0.72–0.80) AUC value in predicting EPE [15]. Additionally, the MSKCC nomogram has higher specificity than the Partin table for predicting EPE. Furthermore, the Nyarangi–Dix normogram is superior to other normograms in terms of net benefit for risk thresholds between 20 and 30% [62]. However, its limitation in practical terms is the fact that the ESUR score must be assessed by a radiologist based on mpMRI, which is not always available. Therefore, in everyday practice, the Soeterik nomogram may prove more useful due to its more accessible combination of parameters (PSAD, clinical stage on MRI, ISUP biopsy grade) with acceptable AUCs ranging from 0.80 to 0.83 in the testing cohort and from 0.77 to 0.78 in the validation cohort [63]. Among all MRI features, the ESUR score and TCCL had the highest AUC and AIC values [62]. In addition, it was shown that the Wibmer nomogram overestimates the risk of EPE for thresholds > 25% [62]. Despite external validation and updates to the Martini nomogram, miscalibration is still present [59]. Nevertheless, it is characterized by sensitivity and specificity compared to mpMRI of 84.2% and 66.7%, respectively [34]. Furthermore, the nomogram reported by Gandaglia et al. is characterized by higher discrimination (71.8% vs. 69.8%, *p* = 0.3 and 71.8% vs. 61.3%, *p* < 0.001) and similar miscalibration and net benefit for probability thresholds above 30% regarding EPE prediction when compared to the MSKCC nomogram and Partin tables [64,65]. Another useful nomogram is the one developed by Sayyid et al., which uses preoperative parameters to assess the risk of side-specific EPE without the use of mpMRI and has a predictive accuracy of 0.74 [66]. An important report is a publication comparing sixteen predictive models, which shows that the models developed by Pak, Patel, Martini, and Soeterik achieved the highest accuracy (AUC ranging from 0.73 to 0.77), adequate calibration for a probability threshold < 40%, and the highest net benefit for a probability threshold > 8% on decision curve analysis [67]. Overall, both traditional and MRI-based nomograms demonstrated moderate accuracy in predicting EPE, but models incorporating mpMRI provide clinically valuable side-specific information for surgical planning. However, limitations related to calibration, overestimation of risk, and restricted availability of MRI-derived parameters still hinder their widespread implementation in routine practice. The most important nomograms are depicted in Table 3 and Table 4.

#### 3.1.5. Alternative Imaging Modalities

##### Micro-Ultrasound

High-resolution micro-ultrasound has emerged as a promising modality for real-time preoperative assessment of extraprostatic extension. Early studies demonstrated high sensitivity and negative predictive value for detecting EPE, including focal microscopic extension, with risk increasing proportionally to the number of micro-ultrasound predictors such as capsular bulging, hypoechoic halo, and obliteration of the vesicle–prostatic angle [79]. A prospective study of 140 patients confirmed that these micro-ultrasound features were strongly associated with non-organ-confined disease, achieving an AUC of 0.88 when combined with clinical parameters [80]. More recently, a large prospective cohort of 391 patients and 612 prostate lobes was used to develop a side-specific micro-ultrasound-based nomogram integrating PSAD, ISUP grade group, maximal core involvement, and MUS-detected EPE, which achieved an internally validated AUC of 0.81, comparable to an mpMRI-based model, while identifying 36% of EPE cases missed by MRI, including lesions invisible on PI-RADS assessment [81]. Finally, a micro-ultrasound-based nomogram developed in a prospective cohort of 295 patients achieved an AUC of 0.77 for micro-ultrasound alone and 0.86 for the multivariable model after internal bootstrap validation [82]. However, it is important to note that after the mentioned studies did not include any external validation, this raises concerns regarding the generalizability of the reported high AUCs to routine clinical practice. Data suggest that micro-ultrasound may complement mpMRI by providing real-time, side-specific risk stratification for nerve-sparing surgical planning, but more studies are needed.

##### Positron Emission Tomography

The use of Prostate-Specific Membrane Antigen Positron Emission Tomography (PSMA PET), particularly in hybrid PSMA PET/MRI imaging, is an advancement in the preoperative staging of prostate cancer, offering a higher sensitivity for detecting EPE compared to mpMRI alone [14,83,84,85]. Meta-analyses indicate that PSMA PET achieved a pooled sensitivity of approximately 0.72 and a high specificity of 0.87, with an overall AUC of 0.87 [83,86]. The intensity of tracer uptake, represented by SUVmax, showed a strong correlation with tumor aggressiveness and Gleason scores, serving as an independent predictor of EPE [83,87]. Despite its high specificity, the method’s sensitivity remains limited by spatial resolution, which may hinder the detection of microscopic EPE [83,86].

Increasing attention has been directed toward multimodal nomograms that integrate metabolic PET parameters, such as SUVmax and PSMA-derived tumor volume (PSMA-TV), with clinical and MRI data [83,87]. Studies have shown that models combining overt EPE on PET, SUVmax ≥ 13.84, and PSMA-TV can achieve an exceptionally high AUC of 0.890 [83]. Integration approaches, which “upgrade” the suspicion of EPE if SUVmax > 12 have demonstrated a significantly high sensitivity—80.4% [85]. Furthermore, recent nomograms utilizing 18F-DCFPyL PSMA-PET/CT with a threshold of SUVmax ≥ 13 have significantly outperformed traditional MRI-only models (AUC 0.754 vs. 0.735) [87]. However, while established clinical and MRI-based nomograms have undergone external validation, the latest PET-integrated models are based on single-center cohorts and require further external validation to ensure their stability in routine clinical practice. The question remains as to whether these exceptionally good results would be maintained after external validation.

#### 3.1.6. Machine Learning Algorithms

An extension of nomograms is algorithms that use machine learning to recognize patterns of EPE based on patient clinical data. The literature confirms their usefulness in predicting EPE; the model cited in the test cohort obtained an area under the receiver operating characteristic curve (AUROC) of 0.81 and an area under the precision–recall curve (AUPRC) of 0.78 [78]. Further evidence is provided by the work of Kwong, J. et al., in which the developed AI model, after external validation, achieved a pooled AUROC of 0.77 and a pooled AUPRC of 0.61 [76]. Side-specific Extraprostatic Extension Risk Assessment tool (SEPERA) predicted side-specific EPE in 68% cases. The biggest defects of the model were false negatives, but none of them were aggressive tumors (grade > 2 or high-risk disease). A recent report is a study combining machine-learning normograms with PSMA-PET, MRI data, and genomics in site-specific EPE prediction [88]. Interestingly, Decipher Genomic Classifier (DGC) scores+PET achieved a better AUC compared to PET+MRI+DGC of 0.85 vs. 0.83, respectively. PET-only predictions were superior to MRI-only predictions; however, multimodal combinations achieved a significant improvement in prediction accuracy. Overall, machine-learning-based nomograms demonstrate similar, and sometimes even better and more flexible performance in EPE prediction compared with traditional models, particularly when combined with multimodal data such as PSMA-PET and genomic classifiers.

### 3.2. Artificial Intelligence in Image Analysis for EPE Prediction

**Figure 1:**
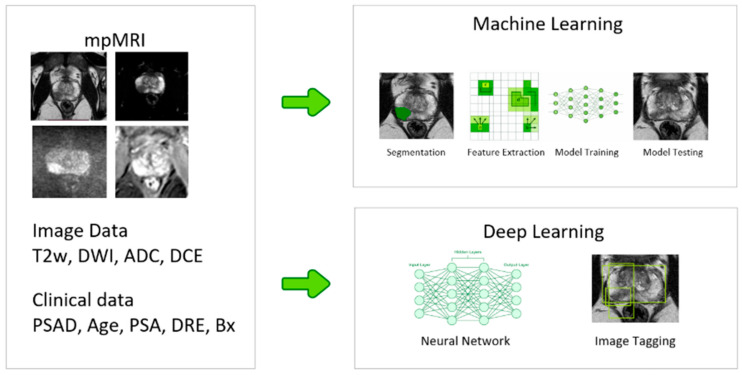
Overview of radiomics-based machine learning versus deep learning approaches for prostate MRI analysis.

Artificial intelligence is increasingly used in medical imaging as a tool to support clinical decisions. In prostate cancer, AI-based image analysis can be divided into two categories. The first uses radiomics combined with classical machine learning, where predefined image features are analyzed. The second focuses on deep learning, which learns patterns directly from images. Simplified differences between those two methods are depicted in Figure 1. The following sections present both approaches.

#### 3.2.1. Machine Learning and Radiomics in Image Analysis for EPE Prediction

Radiomics is a method for analyzing medical images that goes beyond what radiologists can see with their eyes. Instead of just looking at an MRI or CT scan and describing what is visible, radiomics turns these images into large sets of numerical features [89,90]. These features capture detailed information about the shape, texture, intensity, and spatial patterns within the lesion and the nearby tissue [91]. The process starts by segmenting the area of interest, such as the suspected lesion or the prostate capsule, and then extracting features using software like PyRadiomics [92].

After extraction, these features can help build predictive models. They can be used on their own or combined with clinical information, such as PSA levels or Gleason scores [16,93]. In prostate cancer, radiomics is especially valuable since it can detect very subtle changes at the boundary between the tumor and the capsule [16]. These changes may indicate extraprostatic extension or features indicating microscopic extension that are often not visible by eye in standard imaging. The main advantages of radiomics are that it makes image analysis objective, consistent, and measurable, and that it can fit into clinical decision support systems [89,90,91].

An important step in creating radiomic models is selecting features [89,90]. The initial count of features is usually very large, so only the most informative ones are to be kept. This helps reduce the risk of overfitting and makes the model easier to understand. Techniques like recursive feature elimination, mutual information ranking, or regularized regression are often used for this purpose [94,95].

Then, supervised machine learning models are used to predict extraprostatic extension before surgery based on those extracted features. Common algorithms include support vector machines, random forests, and XGBoost [96,97]. Support vector machines handle high-dimensional data well and can differentiate between cases with and without extension [98]. Random forests combine multiple decision trees, which boosts strength and aids in ranking features [97]. XGBoost often achieves high accuracy by modeling complex relationships among variables [96]. In several studies, these models reached areas under the curve between 0.75 and 0.85, especially when trained on carefully chosen radiomic and clinical features [16]. Their main drawback is that they still depend on feature engineering and may struggle with generalizing to other patient populations [99]. Another limitation is the risk of overfitting when applied to small datasets, potentially leading to artificially inflated diagnostic performance [100]. For radiomics, the limitation is that performance relies on image quality, segmentation accuracy, and uniformity of imaging protocols [101]. Despite these challenges, radiomics and ML strategies are important milestones in advanced EPE prediction.

Several studies have already demonstrated the usefulness of radiomics and ML in predicting EPE. Ma et al. developed a T2-weighted MRI-based radiomics signature trained on 210 patients, achieving an AUC of 0.88 in independent validation, clearly outperforming expert radiologists [102]. Damascelli et al. applied a multiparametric MRI radiomic pipeline and demonstrated that a combined T2-weighted and ADC signature was significantly associated with extracapsular extension, reaching an accuracy of 0.84 [103]. Cuocolo et al. created a model based on T2-weighted and diffusion parameters, achieving an AUC of 0.85 in external validation [104]. Fan et al. built mpMRI-based machine-learning models and, using a random-forest classifier, achieved an AUC of 0.85 for predicting EPE [105].

Bai et al. introduced a peritumoral radiomics strategy using a 3–12 mm ring around the tumor and demonstrated better generalizability than intratumoral features, with an external validation AUC of 0.68 [106]. Losnegård et al. analyzed radiomic features derived from the capsule–tumor interface and reported superior diagnostic performance compared with expert radiologists [107].

Xu et al. constructed an mpMRI-based radiomics nomogram integrating radiomic and clinical features, achieving a validation AUC of 0.87 and significantly outperforming the clinical model alone [108]. He et al. evaluated MRI radiomics in a large cohort of 459 patients and showed that the best integrated radiomics–clinical model reached an AUC of 0.73 [109]. Stanzione et al. combined radiomics with the PI-RADS scoring system and demonstrated an incremental benefit over radiological assessment alone [110].

A 2024 meta-analysis summarized all studies available at that time employing radiomics, achieving a pooled AUC of 0.82 [16]. By analyzing these studies, we can conclude that radiomics can identify subtle changes and prostatic extensions more effectively than the visual assessments of radiologists.

Another meta-analysis showed that MRI-inclusive nomograms and AI or radiomics-based models achieved comparable, moderate performance for EPE prediction, suggesting that advanced imaging features do not yet consistently outperform traditional clinical models.

Many studies also merge radiomic features with clinical data like PSA, Prostate Volume, DRE, or biopsy data to create hybrid models, which often outperform models based on imaging or clinical variables alone. However, to date, only four studies employing radiomics and machine-learning methods have adopted this approach [105,106,107,109], reporting AUC values ranging from 0.72 to 0.85.

Another important limitation is the lack of side-specific modeling. As discussed earlier, accurate identification of the side affected by EPE is important for nerve-sparing surgery [7]. Among studies incorporating machine learning and radiomics, the majority predict only the presence of EPE, without specifying the side of involvement [16]. Therefore, future research should build on the strong performance of current radiomics and hybrid radiomics clinical models by developing robust side-specific approaches that translate these methods into personalized tools for surgical decision making.

#### 3.2.2. Deep Learning

Deep learning is a type of artificial intelligence that enables computer systems to learn directly from data like complex medical images. Unlike traditional machine learning approaches, which depend on manually defined and extracted features, deep learning is based on convolutional neural networks that automatically identify patterns within the input data [111,112].

These networks learn in a hierarchical manner, beginning with simple visual characteristics such as edges or signal intensity differences and gradually combining them into more complex anatomical and contextual representations [111,112]. This makes deep learning particularly well-suited for the analysis of multiparametric MRI, where subtle spatial relationships within and around a lesion may carry important diagnostic information that is difficult to capture using conventional image interpretation [113,114].

In recent years, several research groups have applied convolutional neural networks to improve the detection of EPE. Examples include models developed by Hou, Moroianu, Priester, Yao, and Khosravi [115,116,117,118,119]. Together, these studies present a shift toward automated and potentially more flexible prediction systems in prostate cancer imaging, providing an AUC between 0.72 and 0.88 on validation datasets.

It should be noted that side-specific outcome reporting, which is particularly relevant for nerve-sparing surgery, has only been incorporated in a limited number of previously published DL models [115,117,119]. This represents an important gap in the current literature and highlights the need for prediction frameworks that are not only accurate, but also anatomically and surgically relevant.

One of the main advantages of deep learning is that it does not depend on predefined mathematical descriptors or handcrafted image features [113]. Instead, it resembles the way a human observer recognizes visual patterns while offering the ability to analyze large volumes of data and detect image characteristics that may not be visible to the human eye [113]. As a result, deep learning has the potential to make image interpretation more objective and consistent, reduce inter reader variability, and support clinical decision making by contributing to the standardization of radiological assessment [120,121].

Despite these advantages, deep learning also has important limitations. Many models operate as so-called black boxes, meaning that their internal decision-making processes are difficult to interpret from a clinical perspective [122]. This lack of transparency can limit clinician trust and slow the adoption of such tools in everyday practice. In addition, deep learning models are sensitive to variations in imaging data, scanner types, and acquisition protocols. Consequently, models that perform well on internal datasets may show reduced accuracy when applied to external cohorts from different centers [123]. This emphasizes the importance of robust methodological design and external validation. Another major challenge is the requirement for large, high-quality training datasets, which are often difficult to obtain in medical imaging, particularly for highly specific tasks such as predicting extracapsular extension [124].

**Figure 2:**
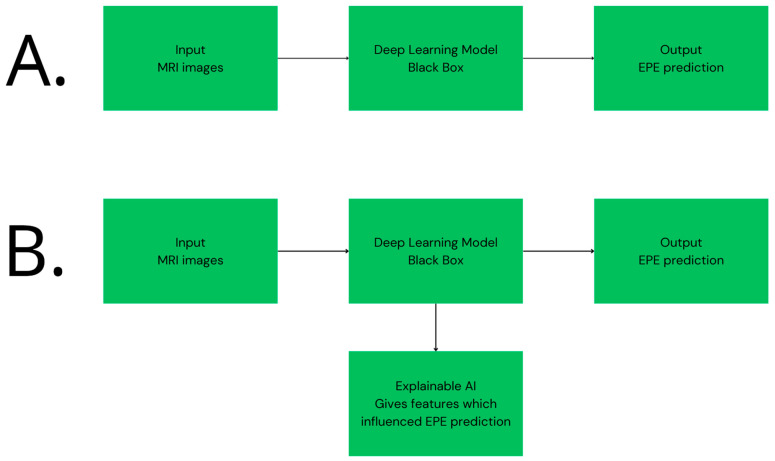
Panel (**A**) shows a conventional deep learning model that generates an EPE prediction from MRI input without insight into the decision process. Panel (**B**) illustrates the same model augmented with explainable AI, providing additional information on the factors contributing to the prediction.

To address these challenges, increasing attention has been directed toward explainable artificial intelligence, the use of larger and more diverse datasets, and the implementation of consistent validation strategies [123,125,126,127]. Explainable artificial intelligence aims to make the decision-making process of complex models more transparent by identifying which image regions or variables contribute most strongly to a given prediction [128]. By linking model outputs to clinically meaningful features, these approaches help align algorithmic reasoning with clinical intuition, which may improve clinician trust and facilitate integration into routine practice [129]. The way explainable AI improves the black box nature of deep learning model prediction is depicted in Figure 2.

Rather than functioning only as probability-generating tools, explainable models provide insight into why a particular prediction is made, allowing clinicians to better understand, evaluate, and contextualize the results [130]. The clinical value of such approaches has already been demonstrated in other areas of medicine, for example, in improving the understanding of hypoxemia risk during anesthesia, where explainability has improved both model transparency and clinical acceptance [131]. With continued methodological advances and access to good datasets, explainable deep learning approaches have the potential to become an important component of precision imaging and personalized treatment planning [114].

In practice, the two approaches—ML and DL are complementary. Classical machine learning can be effective when data volume is limited, and interpretability is important [132,133]. Deep learning becomes advantageous when larger datasets are available and when the diagnostic task benefits from learning complex spatial relationships [132,133]. With the growing adoption of explainable AI and multicenter imaging datasets, both paradigms are increasingly being integrated into clinical decision-support systems, with the shared aim of improving accuracy, consistency, and personalized care. The main characteristics of the two approaches are summarized in Table 5.

#### 3.2.3. AI vs. Specialists

MRI interpreted by an experienced radiologist remains the gold standard for prostate cancer imaging [12]. Artificial intelligence should act as a supportive tool designed to assist and augment radiological decision-making rather than replace the radiologist [134,135]. While AI-based systems may outperform less experienced readers, highly specialized genitourinary radiologists may still achieve better diagnostic accuracy, as depicted in studies [135,136,137]. However, a recent study suggests that newer AI models may outperform even expert radiologists [135]. At present, such evidence remains limited in the available literature. Nevertheless, considering the inter-reader variability of MRI interpretation, AI may serve as a standardizing tool, contributing to more consistent and reproducible assessments across different levels of radiological expertise [135,138].

## 4. Future Directions

An important future direction in the prediction of extraprostatic extension is developing multimodal models that combine imaging features from MRI, biopsy pathology, serum markers like PSA or PSAD, and even genomic classifiers [139,140]. By combining different types of data, multimodal models may improve both sensitivity and specificity, especially in patients with uncertain EPE risks. However, creating these models requires large, well-annotated datasets with synchronized clinical, imaging, and molecular data, which are hard to obtain in most clinical settings [141]. Future studies should focus on building integrated pipelines based on different types of data that can provide personalized risk estimates to guide surgical decisions and on creating well-built datasets.

A significant limitation to clinical adoption of AI-based models for predicting EPE is the lack of external validation and the limited availability of multi-institutional datasets [141,142]. Many models are trained and tested at a single center, often involving similar patient groups, imaging protocols, and annotation standards. This raises concerns about overfitting and poor generalization to real-world clinical scenarios [143,144]. Future efforts must prioritize external validation cohorts with varied demographic and technical characteristics. Collaborative groups, federated learning setups, and publicly available collections of annotated MRI-histopathology pairs could speed up this process. Without strong validation across institutions, clinical use of AI models will probably remain restricted.

Another underexplored area is the direct comparison between artificial intelligence models and expert radiologists, as well as the evaluation of combined strategies in which AI models are used to support human interpretation [145]. While several studies regarding EPE detection have compared model performance with that of radiologists, only a limited number have assessed whether a radiologist assisted by an AI model outperforms either approach alone [115]. Future research should systematically investigate three scenarios: radiologist alone, model alone, and radiologist assisted by the model, to determine whether AI can meaningfully enhance human performance rather than merely replicate it.

The inconsistency in defining ground truth and reporting performance is a major limitation in the current literature on AI for EPE detection. Some studies define EPE based on clinical reports; others use registered histology with precise capsular annotations. This variability in reporting standards complicates comparison and evaluation between models. Protocols like TRIPOD-ML [146,147], QUADAS-AI [148], and the PROBAST-AI [149,150] provide guidelines for good reporting. Adoption of these guidelines would improve reproducibility and help clinicians better evaluate the AI tools in EPE risk assessment.

Precise and reproducible segmentation of the prostate and lesions is essential for radiomics. Manual segmentation takes a lot of time and can vary between experts, making automated segmentation crucial for real-world AI use. Recent advances in 3D architectures like 3D UNet [151], V-Net [152], and nnU-Net [153] have shown strong results in prostate MRI segmentation, often achieving Dice scores above 0.90 [154]. Incorporating such tools into EPE prediction workflows can reduce dependence on operators and improve model scalability.

In addition to the radiologist’s expertise and proper training of AI models, image quality is a critical determinant of EPE detection performance [155,156]. Poor MRI quality may lead to an underestimation of subtle capsular irregularities and compromise both human and algorithmic interpretation. Therefore, future studies should incorporate standardized tools for assessing prostate MRI quality, such as PI-QUAL [157]. Consequently, reporting image quality scores should become a mandatory element of future studies evaluating AI-based EPE prediction models.

Most studies included in this review treated extraprostatic extension as a binary outcome, classifying disease simply as present or absent; however, this approach may be clinically insufficient, as focal capsular breach and established extraprostatic extension carry different prognostic and surgical implications, particularly for nerve-sparing decisions and margin risk stratification [158,159]. Only a limited number of investigations attempted to grade EPE severity [59,61,115,116,117] despite evidence that this distinction provides incremental clinical value [160]. Consequently, binary prediction frameworks may underestimate the biological complexity of periprostatic tumor spread and contribute to discrepancies between reported model performance and real-world surgical outcomes. Future artificial intelligence models should therefore incorporate graded EPE assessment using multi-class or regression-based strategies to better reflect invasion extent and enhance clinical interpretability.

**Figure 3:**
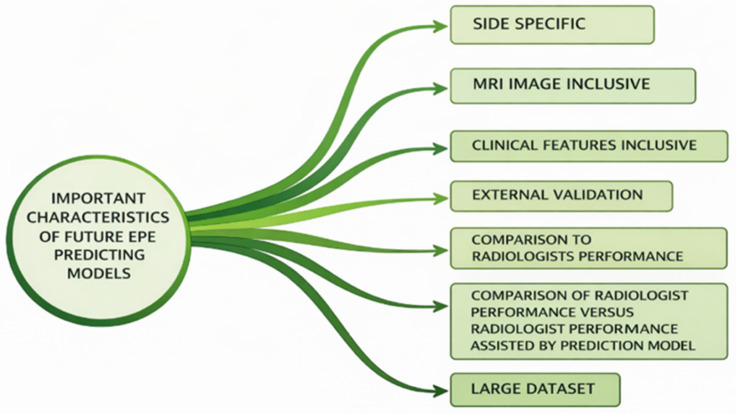
Recommendation of future directions for new EPE predicting models.

The most important features of future EPE prediction models are depicted in Figure 3.

## 5. Conclusions

Preoperative detection of extraprostatic extension is limited by the sensitivity of traditional clinical parameters and mpMRI, as well as by substantial inter-reader variability. These limitations may lead to inappropriate surgical planning with negative consequences for both oncological and functional outcomes.

Radiomics and machine-learning approaches provide more objective and quantitative assessments of mpMRI and match and sometimes even outperform conventional nomograms. In addition, emerging non-MRI imaging modalities, such as high-resolution micro-ultrasound and PSMA PET, may play a complementary role in selected clinical scenarios, particularly when mpMRI findings are equivocal. However, the performance of these advanced approaches remains dependent on image quality, segmentation accuracy, and the heterogeneity of available datasets.

Deep-learning models offer further improvements in analyzing complex imaging patterns and may support more comprehensive, multimodal assessment strategies in the future. Nevertheless, their clinical adoption is still restricted by limited interpretability, insufficient external validation, and the lack of reliable side-specific predictions. Future efforts should therefore focus on developing explainable, multimodal, and externally validated frameworks that integrate complementary imaging tools and artificial intelligence to support personalized surgical decision-making in routine clinical practice.

## Figures and Tables

**Table 1:** Clinical parameters used in EPE prediction.

Parameter	Description	Association with EPE
PSA level	Serum prostate-specific antigen (ng/mL)	Higher PSA → increased risk
Age	Patient age at diagnosis	Older age → higher risk
PSAD	PSA ÷ prostate volume (ng/mL/cc)	Higher density → increased risk
cT stage	Clinical T stage (T1c–T3)	≥T2b/T2c → higher risk
Biopsy Gleason score/grade group	Histologic grade from biopsy	Higher grade → higher risk.
Number of positive cores	Absolute number	More cores → higher risk
% Positive biopsy cores	Positive cores ÷ total cores	Higher % → increased risk
Perineural invasion	Presence on biopsy	Associated with EPE
Lymphovascular invasion	Presence on biopsy	Associated with EPE

**Table 2:** mpMRI parameters used in EPE prediction.

Parameter	Description	Diagnostic Value	Comments
Breach of the capsule	Disruption of the capsule with direct tumor infiltration	Very high specificity	Detects mainly macroscopic EPE
	Outside the prostate		
Tumor-capsule contact length (TCL)	Length of contact between the tumor and the prostate capsule	Increased EPE risk	No standardized threshold
Tumor size	Tumor diameter	Improve sensitivity but reduce specificity	No impact on overall accuracy
ADC entropy	Tissue heterogeneity in MRI	Improve sensitivity but reduce specificity	No impact on overall accuracy
Capsular enhancement sign			
	Early enhancement of the prostate increases diagnostic accuracy		
	Capsule adjacent to the tumor		
Rectoprostatic angle obliteration	The acute angle between the posterior prostate capsule and the anterior rectal wall	Increased EPE risk	
Asymmetry/obliteration of the neurovascular bundle	Abnormal appearance of the NVB	Increased EPE risk	
Periprostatic fat infiltration	Visible tumor tissue projecting into the surrounding periprostatic fat	Increased EPE risk	
Capsular bulging, irregularity,	A contour abnormality of the prostate capsule	Increased EPE risk	The highest diagnostic performance for EPE prediction with clinical parameters
TCA1	Tumor dimensions across two planes	Increased EPE risk	Tested on cT2N0M0 patients
TCA2	Tumor’s contact area within the MRI volume	Increased EPE risk	Tested on cT2N0M0 patients

**Table 3:** Non side-specific normograms.

Normogram	Parameters	AUC Before Validation	AUC—External Validation
Partin tables	PSA, Gleason score, cT stage	0.724 [68]	0.61 [65];
			0.22 [69];
			0.71 [70];
			0.61 [71];
			0.67 [72];
			0.61 [73]
MSKCC	PSA, age, Gleason score	0.71 [9]	0.68 [65];
	cT stage, biopsy cores		0.76 [72];
			0.723 [74];
			0.74 [73]
CAPRA score	PSA, Gleason score, cT stage,	0.66 [57]	0.704 [74]
	% positive cores, age		
Gandaglia without MRI	PSA, ISUP, cT stage,	0.67 [65]	none
	% positive cores		

**Table 4:** Side-specific normograms.

Normogram	Parameters	AUC Before Validation	AUC—External Validation
Martini	PSA, Gleason grade, % core involvement, EPE at MRI	0.821 [58]	0.74 [62];
			0.78 [34];
			0.78 [59];
			0.68 [75];
			0.75 [73]
Gandaglia with MRI	PSA, Gleason grade, % positive cores, EPE at MRI, max. diameter of the index lesion at MRI	0.70 [65]	none
	PSA, Gleason grade, % positive cores, EPE at MRI, max. diameter of the index lesion at MRI		
Wibmer	Age, PSA, PSAD, ISUP, % positive cores, max. tumor extent, PIRADS, max. Lesion Diameter, Length of Capsular Contact, Presence of EPE	0.828 [61]	0.72 [62]
	Age, PSA, PSAD, ISUP, % positive cores, max. tumor extent, PIRADS, max. lesion diameter, length of capsular contact, presence of EPE		
Nyarangi-Dix	PSA, cT stage, prostate volume, ISUP, ESUR criteria, capsule contact length	0.87 [60]	0.76 [62]
Soeterik	PSAD, clinical stage on MRI, ISUP	0.77–0.83 [63]	0.75 [62];
			0.80 [60];
			0.69 [76];
			0.80 [77];
			0.81 [73]
Sayyid	Age, PSA, prostate volume, palpable nodule on DRE, hypoechoic nodule on TRUS, max. core involvement, % positive cores, ISUP	0.74 [66]	0.75 [78];
			0.75 [76];
			0.77 [73]
	Age, PSA, prostate volume, palpable nodule on DRE, hypoechoic nodule on TRUS, max. core involvement, % positive cores, ISUP		

**Table 5:** Comparison of basic features of Radiomics + Machine Learning models with Deep Learning Models.

Category	Radiomics + Machine Learning	Deep Learning
Feature extraction	Based on handcrafted features extracted after segmentation.	Features are learned automatically from raw images.
Workflow complexity	Requires multiple steps: segmentation, feature extraction, feature selection, and modelling.	Features are learned automatically from raw images.
Explainability	Relatively transparent, features can be interpreted clinically.	Often, a black box requires explainable AI methods for interpretation.
Data dependency	It can be applied with limited datasets, but it is sensitive to feature engineering.	Requires large, well-annotated datasets to perform reliably.

## Data Availability

No new data were created or analyzed in this study.

## Abbreviations

The following abbreviations are used in this manuscript:

EPE	Extraprostatic extension
mpMRI	Multiparametric Magnetic Resonance Imaging
ML	Machine learning
DL	Deep learning
PSA	Prostate-specific antigen
PSAD	PSA density
ISUP	International Society of Urological Pathology
TNM	Tumor, Node, Metastasis
EAU	European Association of Urology
csPC	Clinically significant prostate cancer
PC	Prostate cancer
SVI	Seminal vesicle invasion
NVB	Neurovascular bundle
TCA1	Tumor contact area 1
TCA2	Tumor contact area 2
TCL	Tumor contact length
MSKCC	Memorial Sloan Kettering Cancer Center
CAPRA	Cancer of the Prostate Risk Assessment
AUROC	Area under receiver operating characteristic curve
AUPRC	Area under precision–recall curve
SEPERA	Side-specific Extraprostatic Extension Risk Assessment
DGC	Decipher Genomic Classifier
PET	Positron emission tomography
PSMA	Prostate specific membrane antigen
PSMA-PET	Prostate-specific membrane antigen positron emission tomography
PSMA-TV	Prostate-specific membrane antigen–derived tumor volume
SUVmax	Maximum standardized uptake value
TV	Tumor volume
